# Association between serum LH levels on hCG trigger day and live birth rate after fresh embryo transfer with GnRH antagonist regimen in different populations

**DOI:** 10.3389/fendo.2023.1191827

**Published:** 2023-07-05

**Authors:** Ruiqiong Zhou, Mei Dong, Li Huang, Xiulan Zhu, Jinyan Wei, Qianyu Zhang, Dun Liu, Xiqian Zhang, Fenghua Liu

**Affiliations:** Center for Reproductive Medicine, Guangdong Women and Children Hospital, Guangzhou, Guangdong, China

**Keywords:** GnRH antagonist, live birth rate, controlled ovarian stimulation (COS), ovarian response, luteinizing hormone (LH)

## Abstract

**Objective:**

To investigate whether serum LH levels on hCG trigger day are associated with live birth rate (LBR) after fresh embryo transfer with GnRH antagonist regimen in different populations.

**Methods:**

This study was a retrospective study. A total of 3059 fresh embryo transfers were divided into three populations: predicted normal ovarian responders (NOR) (n=2049), patients with PCOS (n=533), and predicted poor ovarian responders (POR) (n=477). Each population was stratified into three groups based on LH levels: < 25th percentile, 25–75th percentile, and > 75th percentile. The primary outcome of the study was LBR, and secondary outcomes included implantation, clinical pregnancy, and early pregnancy loss rates. Univariable and multivariable regression analyses were performed to adjust for potential confounders.

**Results:**

In NOR, compared to the reference group (>75th percentile), LBR was significantly lower in the < 25th percentile group (adjusted OR=0.662; 95%CI, 0.508-0.863) and 25-75th percentile group (adjusted OR=0.791; 95%CI, 0.633-0.988). In PCOS patients, LBR decreased significantly in the < 25th percentile group (41.4%) compared to the 25-75th percentile group (53.7%) and > 75th percentile group (56.1%). In addition, the LBR was lower in the < 25th percentile group (33.6%) compared with the 25-75th percentile group (43.4%) and the>75th percentile group (42.0%) in POR, but this was not statistically significant.

**Conclusions:**

High serum LH levels are associated with increased LBR after fresh embryo transfer in GnRH antagonist cycles, which may be attributable to higher implantation rate. LH may be a predictor of whether to schedule fresh embryo transfer in IVF cycles for better clinical outcomes.

## Introduction

Over the past decade, gonadotropin-releasing hormone (GnRH) antagonist regimen has emerged as one of the leading controlled ovarian hyperstimulation (COH) regimens due to its comparable convenience, safety, and efficacy compared to GnRH agonist (1–3). The influence of GnRH antagonist on clinical outcomes of fresh embryo transfer has become a matter of debate (1, 3). Although some studies have not found significant difference in pregnancy outcomes between GnRH antagonists and agonists, others have reported that GnRH antagonists were associated with lower clinical pregnancy and live birth rates after fresh embryo transfer compared with GnRH agonists (4–8). A meta-analysis showed that in the general IVF population, when patients with polycystic ovary syndrome (PCOS) or poor responders were excluded, GnRH antagonists were associated with lower ongoing pregnancy rate compared to agonists (9).

Although the ideal number of oocytes and embryos can be obtained with COH, the implantation rate after fresh embryo transfer is still relatively low in GnRH antagonist cycles (1, 3). An important issue regarding the use of GnRH antagonist is the inability to predict factors that may affect pregnancy outcomes in fresh IVF cycles, which is critical in deciding whether fresh embryo transfer should be selected.

It has been demonstrated that LH not only plays an important role in follicle development, ovulation, and steroidogenesis, but also affects luteal function and endometrial development (10, 11). GnRH antagonist cause a rapid and profound inhibition of endogenous LH secretion, which occurs when follicle and endometrium development are most sensitive to LH activity (12–14). The function of LH in the ovary by binding to LH/choriogonadotropin receptor (LHCGR) is well known (15, 16). Previous studies have identified LHCGR expression in the uterus, suggesting that LH may affect endometrial receptivity and placentation (17, 18). However, the specific mechanism by which LH affects implantation process remains largely unknown.

The question of whether LH concentrations in GnRH antagonist cycles are associated with pregnancy outcomes after fresh embryo transfer remains relatively sparse and controversial. Previous studies have identified the possible effects of premature LH increase as a sign of premature luteinization, which may lead to reduced oocyte yield and poor embryo implantation as a result of elevated progesterone (19, 20). Nonetheless, LH levels during stimulation are not always associated with progesterone elevation, which may be related to inconsistent findings on whether it affects clinical outcomes (21). Some studies showed that LH levels did not affect clinical outcomes (22–24), and yet others found that low LH levels were associated with adverse pregnancy outcomes (25–28). These controversial results may be related to the heterogeneity of the populations enrolled in different studies, and various control methods for confounders among studies, which require further research to clarify.

The aim of this study was to investigate the association between LH levels on hCG trigger day and live birth rate (LBR), with a particular focus on different populations.

## Materials and methods

### Study design and participants

This study was a retrospective study performed at the Reproductive Medicine Center of Guangdong Women and Children’s Hospital. The Institutional Review Board of the hospital has approved the study protocol. This study included patients undergoing their first fresh embryo transfer using GnRH antagonist regimen between January 2014 and October 2020. The inclusion criteria were as follows: (i) maternal age ≤ 40 years; (ii) day-3 fresh embryo transfer; (iii) at least one embryo was available. The exclusion criteria were as follows: (i) uterine abnormalities and intrauterine adhesion; (ii) endometrium thickness on hCG trigger day < 7 mm; (iii) recurrent spontaneous abortion; (iv) hypothalamic or pituitary amenorrhea (29, 30); (v) core data missing. In this study, we conducted separate analyses of three populations: predicted normal ovarian responders (NOR), patients with PCOS and predicted poor ovarian responders (POR). PCOS was defined as patients who met two of the three criteria (oligo- and/or anovulation, hyperandrogenism, and PCOM) according to the revised Rotterdam Consensus (31). POR were considered: antral follicle count (AFC) < 5 or AMH < 1.1 ng/ml or previous adverse ovarian response. NOR were patients with normal ovarian reserve and regular menstrual cycle (9).

### Ovarian stimulation

A flexible GnRH antagonist regimen was used for ovarian stimulation. In brief, recombinant follicle-stimulating hormone (Gonal-f; Merck Serono or Puregon; MSD, Organon) at a dose of 100 to 300 IU per day was administered on day 2 or 3 of the menstrual cycle. The doses were adjusted according to ovarian response assessed by ultrasound and measurement of serum hormone levels every 3 to 4 days. The GnRH antagonist (Ganirelix; MSD, Organon) was started when at least one follicle was ≥ 12 mm at a daily dose of 0.25 mg and continued until the day of hCG trigger. When at least three follicles measured 17 mm or at least two follicles reached 18 mm in diameter, hCG was administered at a dose of 6000 to 10000 IU to induce oocyte maturation. Oocyte retrieval was performed 35-36 hours later by transvaginal ultrasound-guided follicle aspiration, and oocytes were fertilized by either IVF or ICSI depending on sperm quality. Serum LH, estradiol (E2) and progesterone (P) levels were measured using an electrochemiluminescence immunoassay (Roche Diagnostics Inc., Germany) on the Roche Elecsys 2010 automated immunoassay analyser.

### Embryo transfer and luteal phase support

On the third day after oocyte retrieval, a maximum of two embryos were routinely transferred. A good-quality embryo was defined as day 3 embryos with < 20% fragmentation, and regular-sized cells. Freeze-all strategy was performed in patients at high risk for ovarian hyperstimulation syndrome, abnormal endometrial morphology or thickness (e.g., endometrium thickness < 7 mm), and serum P levels ≥ 1.5 ng/ml during COH.

The luteal phase support was started one day after oocyte retrieval. Intramuscular progesterone (40 mg once daily) or a combination of vaginal progesterone sustained-release gel (Crinone 8%, 90mg once daily) and oral progesterone (Dydrogesterone, 10 mg twice daily) was administered until 10 weeks of gestation. The method of progesterone supplementation depends on patient preference, as there is no clear medical evidence that using one regimen is better than another (32–34).

### Outcome measures

The primary outcome of the study was LBR, which was defined as the delivery of a live infant after 24 gestational weeks. The secondary outcomes included implantation rate (number of intrauterine sacs divided by number of embryos transferred), clinical pregnancy (presence of at least one gestational sac in the uterine cavity at 5 weeks after embryo transfer), and early pregnancy loss (spontaneous loss of clinical pregnancy before 12 weeks of gestation). Cycle outcomes included oocyte yield (ratio of the number of oocytes retrieved to the number of follicles with an average diameter >10 mm on hCG trigger day), normal fertilization rate (ratio of the number of two pronuclear fertilized eggs to the number of oocytes for insemination), usable cleavage embryo rate (ratio of available embryos to cleavage embryos on day 3), and good-quality embryo rate (ratio of good-quality embryos to normally fertilized cleavage embryos on day 3).

### Statistical analysis

All statistical analyses were performed using the statistical software package (SPSS, version 22.0). We used the Kolmogorov–Smirnov test to determine whether continuous variables were normally distributed. Continuous variables were presented as mean with standard deviation (mean ± SD) or median with interquartile range (median (Q1, Q3)), and categorical variables were described as number with percentage. The variables between live birth and non-live birth groups were compared using Student’s t-test or Mann-Whitney U test, and Chi-square or Fisher’s exact test, as appropriate. *P-*value < 0.05 was considered statistically significant.

We then performed separate analyses of three populations: NOR, patients with PCOS, and POR. Univariable and multivariable logistic regression analyses were used to identify potential confounding factors that may be independently associated with live birth for three populations. Confounding factors were assessed by univariable analysis and then added into multivariable regression model for adjustment. In multivariable models, variables with significance in the univariable analysis at *P* < 0.10 or more and variables that may potentially have an effect on live birth (e.g., body mass index (BMI)) were included. To assess the impact of LH level on the incidence of clinical outcomes, first, univariable and multivariable regression analyses were performed in three populations when the variable LH level on hCG trigger day was used as a continuous variable to adjust for confounders (**Supplementary Tables 1**, **3**). Then, each population was stratified into three groups according to the interquartile range of LH levels on hCG trigger day: < 25th percentile group, 25–75th percentile group, and > 75th percentile group. Using the LH > 75th percentile group as the reference group, adjusted odds ratio (OR) and 95% confidence interval (CI) for LBR in other categories were calculated.

## Results

### Study population

**Figure 1:**
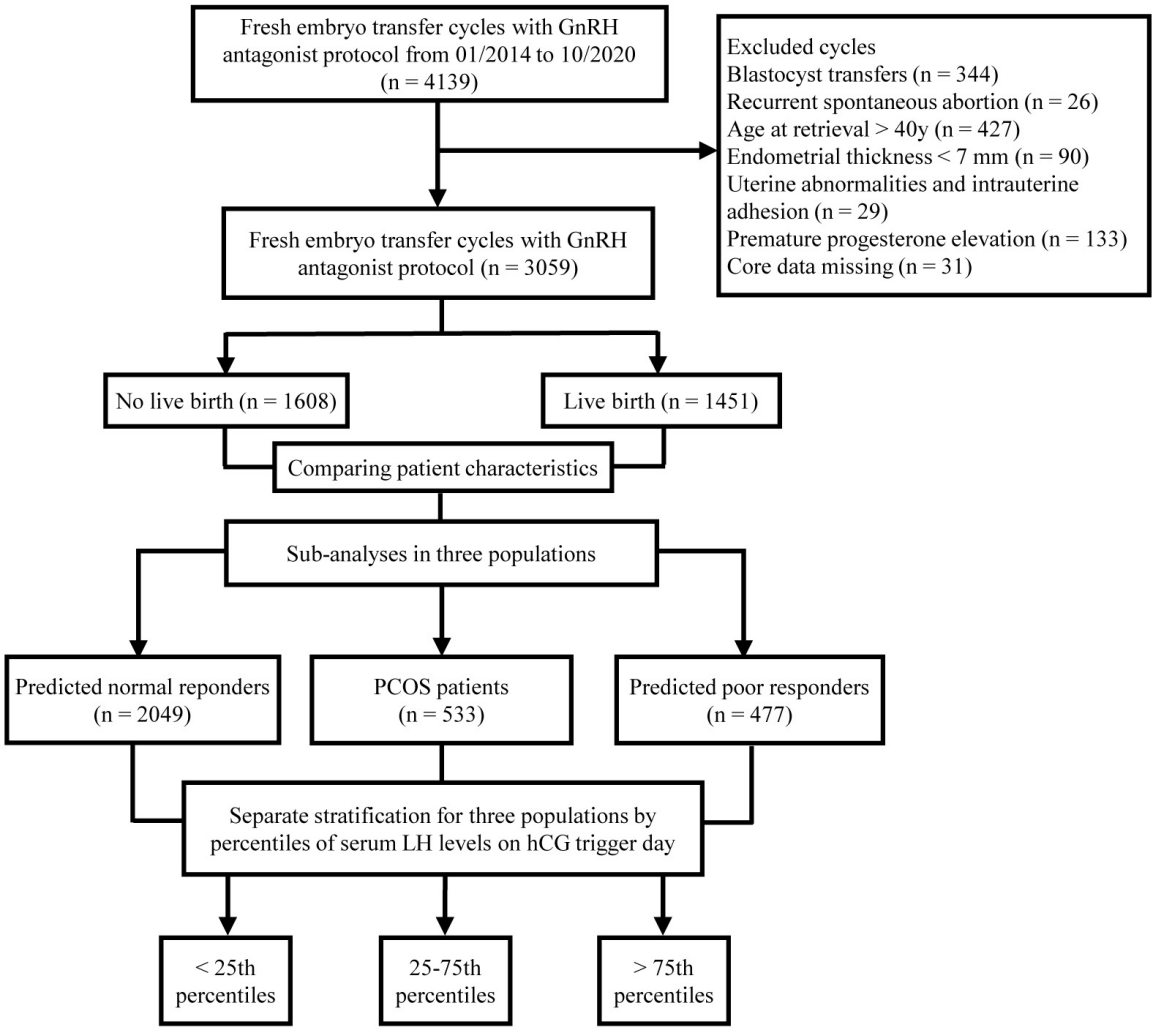
Flow chart of the study.

A total of 3059 patients who met the inclusion and exclusion criteria were included in this study. The flowchart of the study is shown in **Figure 1**. Among them, 1451 patients achieved live births, while 1608 patients did not achieve live births after fresh embryo transfer. Baseline characteristics were compared between patients who did and did not achieve live birth (**Table 1**). There were significant differences in terms of age, AFC, gonadotropin dose, days of stimulation, number of embryos transferred, rate of good-quality embryos transferred, and endometrial thickness on hCG trigger day between the two groups. Notably, serum LH levels on hCG trigger day were significantly higher in patients achieved live births than those of non-live births, while E2 and P levels did not show any significant differences (**Table 1**).

**Table 1:** A comparison of baseline characteristics according to whether patients achieved a live birth after fresh embryo transfer.

Parameters	No live birth (n = 1608)	Live birth (n = 1451)	P value
Age (years)	32.75 ± 4.62	31.31 ± 4.11	< 0.001
BMI (kg/m^2^)	22.10 ± 3.11	22.01 ± 3.11	0.289
AFC	11.78 ± 7.64	13.21 ± 7.87	< 0.001
Basal FSH	7.53 ± 2.64	7.44 ± 2.67	0.081
Basal LH	5.74 ± 3.40	5.83 ± 3.36	0.180
Gonadotropin dose	2034 ± 773	1980 ± 753	0.049
Days of stimulation	9.89 ± 2.08	10.10 ± 2.22	0.012
Antagonist dose	1.22 ± 0.66	1.25 ± 0.71	0.244
Days of antagonist	4.85 ± 2.47	4.92 ± 2.58	0.365
r-LH supplementation	41 (2.5)	30 (2.1)	0.376
Hormone levels on hCG trigger day			
LH	3.35 ± 2.52	3.62 ± 2.85	0.002
Estradiol	2347.7 ± 1263.5	2410.5 ± 1255.8	0.093
Progesterone	0.74 ± 0.33	0.72 ± 0.32	0.122
ICSI treatment	451 (28.0)	383 (26.4)	0.306
No. of embryos transferred			< 0.001
Single	261 (16.2)	112 (7.7)	
Double	1347 (83.8)	1339 (92.3)	
Rate of good-quality embryos transferred	2277/2955 (77.1)	2309/2790 (82.8)	< 0.001
Endometrial thickness (mm) *	10.30 ± 2.12	10.72 ± 2.07	< 0.001
Route of progesterone supplementation			0.975
Intramuscular	975 (60.6)	880 (60.7)	
Vaginal + oral	633 (39.4)	570 (39.3)	

Continuous variables are presented as mean ± SD or median (Q1, Q3). Categorical variables are presented as number (percentage).

*The day of hCG administration. BMI, body mass index; AFC, antral follicle count.

To investigate the relationship between LH levels and LBR in different populations, we divided patients into three categories: NOR (n=2049), those with PCOS (n=533) and POR (n=477). First, univariable and multivariable regression analyses were performed in three populations when the variable LH level on hCG trigger day was used as a continuous variable to adjust for confounders (**Supplementary Tables 1**, **3**). Then, each population was stratified into three groups according to the interquartile range of LH levels on hCG trigger day. The interquartile range (25th to 75th) for LH in NOR was 1.62 to 3.86 mIU/ml; 2.25 to 5.68 mIU/ml in PCOS patients; and 2.14 to 4.72 mIU/ml in POR (**Table 2**).

**Table 2:** Pregnancy outcomes after fresh embryo transfer according to LH stratification on hCG trigger day in different populations.

Pregnancy outcomes according to LH stratification				
Subgroups	Live birth	Implantation rate	Clinical pregnancy	Early pregnancy loss
Normal responders				
< 25th	229/510 (44.9) ^a^	361/973 (37.1) ^a^	267/510 (52.4) ^a^	18/267 (6.7)
25-75th	486/1028 (47.3) ^a^	793/1942 (40.8) ^a^	578/1028 (56.2) ^a, b^	60/578 (10.4)
> 75th	269/511 (52.6) ^b^	431/958 (45.0) ^b^	309/511 (60.5) ^b^	29/309 (9.4)
*P* value	0.037	0.002	0.033	0.241
PCOS patients				
< 25th	55/133 (41.4) ^a^	104/257 (40.5) ^a^	72/133 (54.1) ^a^	5/72 (6.9)
25-75th	144/268 (53.7) ^b^	256/507 (50.5) ^b^	178/268 (66.4) ^b^	14/178 (7.9)
> 75th	74/132 (56.1) ^b^	135/248 (54.4) ^b^	95/132 (72.0) ^b^	9/95 (9.5)
*P* value	0.029	0.004	0.007	0.826
Poor responders				
< 25th	39/116 (33.6)	69/216 (31.9)	52/116 (44.8)	9/52 (17.3)
25-75th	105/242 (43.4)	163/439 (37.1)	125/242 (51.7)	18/125 (14.4)
> 75th	50/119 (42.0)	74/205 (36.1)	59/119 (49.6)	7/59 (11.9)
*P* value	0.2	0.421	0.481	0.717

Variables are presented as number (percentage). ^a,b^ Different superscripts within the same line means statistically difference between subgroups.

The interquartile range (25th to 75th) for LH in normal responders was 1.62 to 3.86 mIU/ml; 2.25 to 5.68 mIU/ml in PCOS patients; and 2.14 to 4.72 mIU/ml in poor responders.

### Predicted normal ovarian responders

In NOR, serum LH level on hCG trigger day as a continuous variable was positively associated with live birth in univariable analysis (OR=1.042; 95%CI, 1.002-1.083) (**Supplementary Table 1**). Pregnancy outcomes were then subdivided into three groups by LH stratification, as presented in **Table 2**. The LBR was significantly lower in the < 25th percentile (44.9%) and 25-75th percentile group (47.3%) than that of the>75th percentile group (52.6%). There was a significantly lower implantation rate for the < 25th percentile group (37.1%) and 25-75th percentile group (40.8%) compared with the>75th percentile group (45%). The clinical pregnancy rate was also found to be significantly lower in the < 25th percentile group than the>75th percentile group (52.4% vs. 60.5%). However, there was no significant difference in early pregnancy loss rate. Additionally, cycle outcomes including oocyte yield, normal fertilization rate, rate of usable cleavage embryos, and rate of good-quality embryos did not differ significantly between three groups (**Supplementary Table 2**).

**Figure 2:**
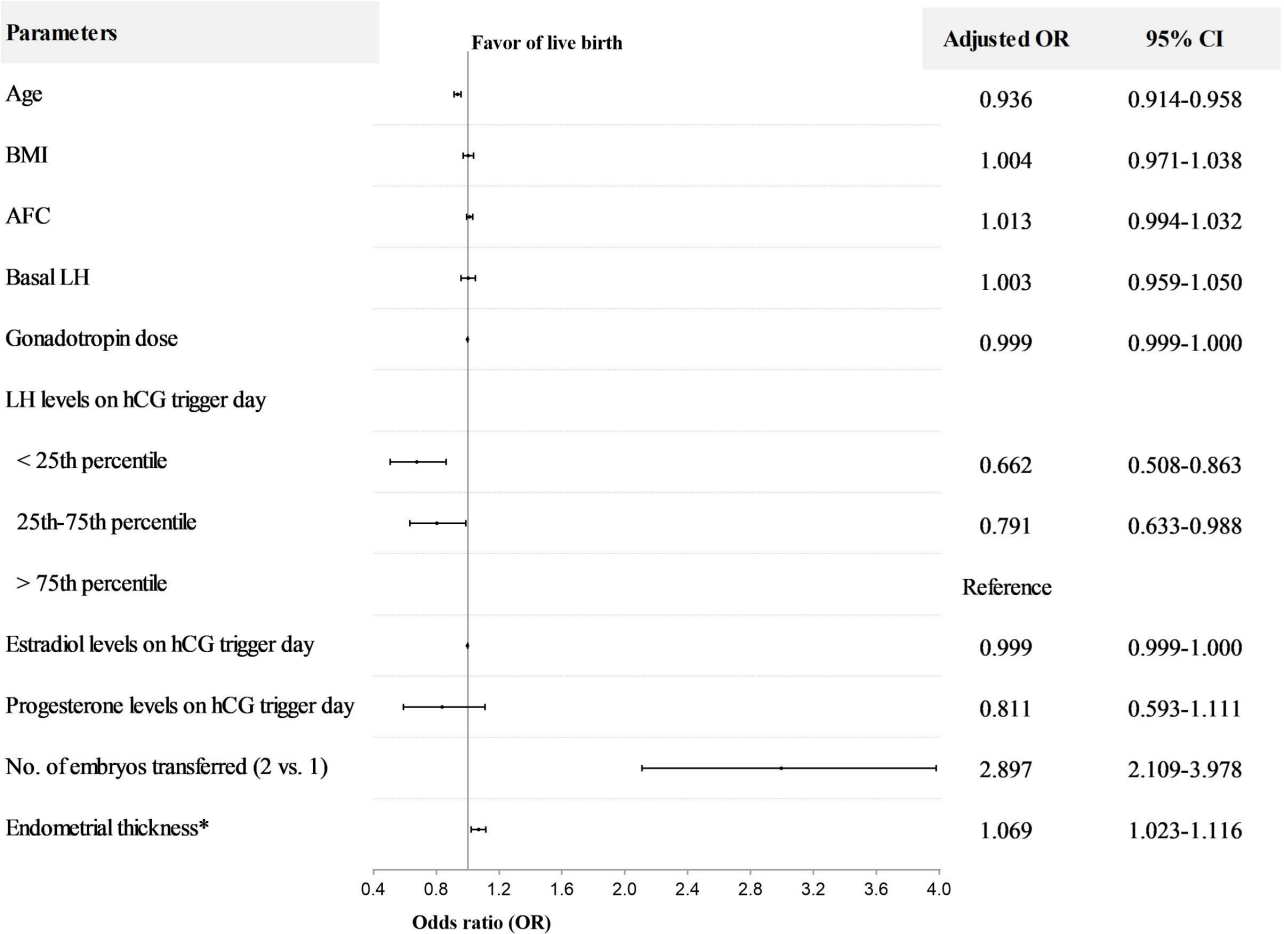
Multivariable regression analysis for live birth after fresh embryo transfer in normal responders. *The day of hCG administration. OR, odds ratio; CI, confidence interval; BMI, body mass index; AFC, antral follicle count. The interquartile range (25th to 75th) for LH was 1.62 to 3.86 mIU/ml in normal responders.

After controlling for age, BMI, AFC, basal LH, gonadotropin dose, E2 and P levels on hCG trigger day, number of embryos transferred, and endometrial thickness on hCG trigger day, LH levels on hCG trigger day as a continuous variable were positively associated with live birth (OR=1.060; 95%CI, 1.016-1.105) (**Table 3**). Furthermore, when LH was stratified as a categorical variable, multivariable analysis revealed that LBR significantly decreased in the < 25th percentile group (adjusted OR=0.662; 95%CI, 0.508-0.863) and 25-75th percentile group (adjusted OR=0.791; 95%CI, 0.633-0.988), compared to the>75th percentile group (**Figure 2**). The other variables with a significant impact on live birth were age (adjusted OR=0.936; 95%CI, 0.914-0.958), number of embryos transferred (adjusted OR=2.897; 95%CI, 2.109-3.978) and endometrial thickness on hCG trigger day (adjusted OR=1.069; 95%CI, 1.023-1.116).

**Table 3:** Multivariable regression analysis for live birth after fresh embryo transfer for different populations.

Parameters	Normal responder Adjusted OR (95% CI)	PCOS Adjusted OR (95% CI)	Poor responder Adjusted OR (95% CI)
Age	0.937 (0.915-0.959)	0.978 (0.931-1.028)	0.900 (0.857-0.945)
BMI	1.005 (0.972-1.039)	0.933 (0.881-0.988)	1.009 (0.935-1.089)
AFC	1.012 (0.993-1.031)	0.999 (0.968-1.031)	0.927 (0.856-1.005)
Basal LH	1.004 (0.959-1.050)	0.964 (0.930-0.999)	0.995 (0.917-1.079)
Gonadotropin dose	0.999 (0.999-1.000)	1.000 0.999-1.000)	0.999 (0.999-1.000)
LH levels on hCG trigger day	1.060 (1.016-1.105)	1.084 (1.022-1.149)	1.027 (0.961-1.099)
Estradiol levels on hCG trigger day	0.999 (0.999-1.000)	0.999 (0.999-1.000)	1.000 (0.999-1.000)
Progesterone levels on hCG trigger day	0.790 (0.577-1.081)	0.908 (0.508-1.626)	0.739 (0.355-1.541)
No. of embryos transferred (2 vs. 1)	2.925 (2.129-4.020)	1.027 (0.562-1.878)	2.301 (1.335-3.968)
Endometrial thickness *	1.069 (1.024-1.116)	1.138 (1.040-1.246)	1.036 (0.938-1.144)

*The day of hCG administration. OR, odds ratio; CI, confidence interval; BMI, body mass index; AFC, antral follicle count.

### Women with PCOS

**Figure 3:**
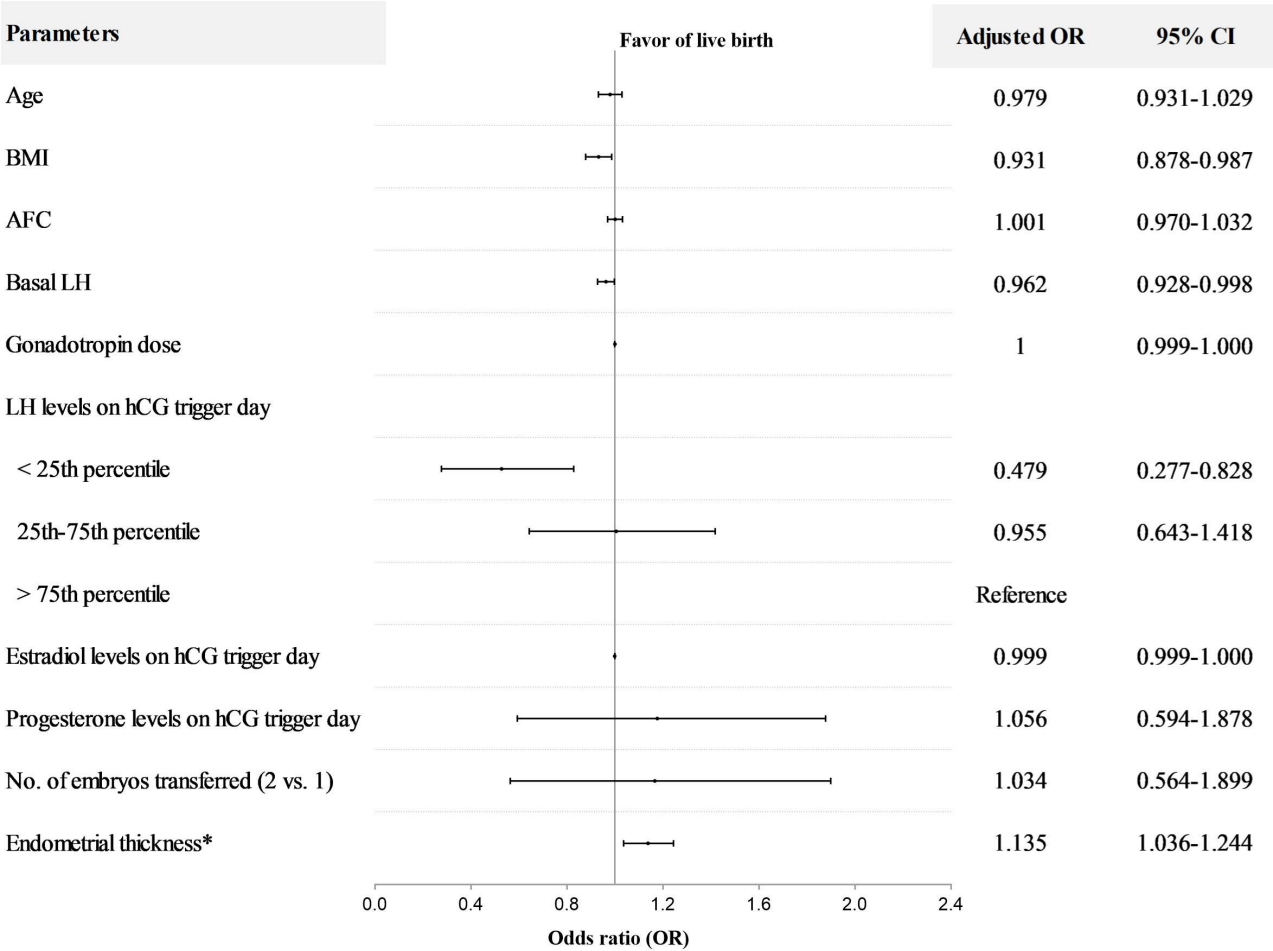
Multivariable regression analysis for live birth after fresh embryo transfer in PCOS patients. *The day of hCG administration. OR, odds ratio; CI, confidence interval; BMI, body mass index; AFC, antral follicle count. The interquartile range (25th to 75th) for LH was 2.25 to 5.68 mIU/ml in PCOS patients.

In PCOS patients, LH level on hCG trigger day as a continuous variable was positively associated with live birth in both univariable (OR=1.066; 95%CI, 1.011-1.124) and multivariable analyses (adjusted OR = 1.084; 95% CI, 1.022–1.149), with results similar to those of NOR (**Supplementary Tables 1**, **3**). The PCOS patients were also stratified into three groups by LH stratification. The LBR in the < 25th percentile group (41.4%) was significantly lower than the 25-75th percentile group (53.7%) and >75th percentile group (56.1%). In addition, both implantation and clinical pregnancy rates decreased significantly in the < 25th percentile group compared to the 25-75th percentile group and >75th percentile group (**Table 2**). The incidence of early pregnancy loss did not differ among the three groups. In terms of cycle outcomes, the rate of good-quality embryos in the < 25th percentile group (76.4%) was significantly lower than the 25-75th percentile group (80.4%) and >75th percentile group (82.1%), while oocyte yield, normal fertilization rate, and rate of usable cleavage embryos did not show any difference between groups (**Supplementary Table 2**). After adjusting for confounders, the LBR decreased significantly in the < 25th percentile group compared with the>75th percentile group (adjusted OR=0.479; 95%CI, 0.277-0.828) (**Figure 3**). Moreover, for PCOS patients, BMI (adjusted OR=0.931; 95%CI, 0.878-0.987), basal LH (adjusted OR=0.962; 95%CI, 0.928-0.998), and endometrial thickness on hCG trigger day (adjusted OR=1.135; 95%CI, 1.036-1.244) were also independent predictors of live birth in multivariable model (**Figure 3**).

### Predicted poor ovarian responders

In patients with POR, no evidence of a statistical association between LH levels as a continuous variable and live birth was found in both univariable (OR=1.008; 95%CI, 0.949-1.072) and multivariable analyses (adjusted OR=1.027; 95%CI, 0.961-1.099) (**Supplementary Tables 1**, **2**). However, the LBR appeared to be lower in the < 25th percentile group (33.6%) compared with the 25-75th percentile group (43.4%) and the>75th percentile group (42.0%), but this was not statistically significant (**Table 2**). Cycle outcomes showed that oocyte yield decreased significantly in the < 25th percentile group (89.7%) compared with the 25-75th percentile group (93.4%) and>75th percentile group (92.8%). Nonetheless, other cycle outcomes did not present any significant difference among groups (**Supplementary Table 2**). In addition, controlling for major covariates, age was inversely associated with live birth (adjusted OR = 0.900; 95% CI, 0.857–0.945), whereas the number of embryos transferred was positively associated with live birth in POR (adjusted OR = 2.301; 95% CI, 1.335–3.968) (**Table 3**).

## Discussion

This study investigated the impact of serum LH levels on hCG trigger day on LBR after fresh embryo transfer in consideration of different populations for the first time. The absolute risk reduction of LBR was 7.7% in NOR and 14.7% in PCOS patients when comparing the low LH group with the high LH group. In POR, the low LH group appeared to have a lower LBR than the middle and high LH groups, but not significantly, which may be related to insufficient patients enrolled.

A few available studies have investigated the effect of LH levels on LBR after fresh embryo transfer with GnRH antagonist regimen. However, previous studies have yielded conflicting results. Contrary to our findings, Marviel et al. showed no significant difference in clinical outcomes between different LH levels on the day of hCG administration, divided into two groups based on an arbitrary threshold of 0.5 IU/L (n=270) (22). In their study, potential bias was reduced by only selecting relatively homogenous population without adjusting for confounders that could have had impact on clinical outcomes. Similarly, Griesinger et al. reported that LH concentrations on day 8 of stimulation were not associated with ongoing pregnancy rates (24). Their retrospective study included 1764 patients pooled from six clinical trials with different purposes, which resulted in a huge heterogenous population. Many confounders affecting pregnancy rate may not have been adequately accounted for, including endometrial thickness and the number of embryos transferred. The study by Luo et al. was consistent with our conclusion, and their results showed that low LH significantly reduced LBR after fresh embryo transfer (38.0% vs. 51.5%) (28). They included 1480 normogonadotropic women underwent COH, and arbitrarily divided patients into low and high LH groups with a cutoff of 4 IU/L, which may not be an appropriate threshold for stratification. In a retrospective study of 619 cycles, Chen et al. found that LH ≤ 0.8 mIU/ml during COH was associated with higher early pregnancy loss rate, but no significant difference in implantation and live birth rates (26). Most previous studies have failed to adequately consider different populations, and they used an arbitrary LH threshold for grouping. Some studies included small sample sizes or did not use LBR as the primary outcome, which may not be sufficient to draw valid conclusions.

The present study provides the largest (n=3059) and well-controlled analysis of the relationship between LH levels and LBR after fresh embryo transfer. The particular strength of this study is that it took into account patient types as comprehensively as possible, as well as the presence of various potential confounders. Great efforts were taken to minimize sources of bias, particularly through the use of univariable and multivariable regression models. Unlike previous studies, which either only included normogonadotropic women or did not differentiate between different populations, the present study aims to investigate the association between LH levels and LBR in three populations: NOR, patients with PCOS and POR. Confounding factors were also minimized by making each population more homogeneous. Most studies used only a single LH value to arbitrarily define low and high LH, which was insufficient to reflect the distribution of LH levels across the population, and statistical difference may be compromised when choosing inappropriate LH level for stratification. In our study, LH levels were used as both continuous and categorical variables to examine the effect of LH levels on clinical outcomes in three populations. We stratified different populations using the 25th and 75th percentiles of LH levels, respectively, and tried to find an appropriate LH cutoff for each population, which might be more applicable to clinical practice. Nowadays, clinicians are more familiar the “one size does not fit all” concept. The analyses of different populations provide new perspectives to objectively consider different treatments for personalized patient characteristics, thereby may contribute to better clinical outcomes.

The present study has certain limitations due to its retrospective design. The large sample size of this study may partially reduce selection and statistical bias, nevertheless, the numbers of PCOS and POR patients remained small, which may potentially affect statistical efficiency. In the present study, we did not find significant deleterious effect of LH levels on oocyte performance. However, we only included fresh transfer cycles to analyze oocyte performance and embryonic development, so the results should be interpreted with caution. When progesterone levels were ≥ 1.5 ng/ml during stimulation, we use freeze-all strategy to rule out adverse effect of premature luteinization on fresh IVF outcomes, based on previous studies (35–38). However, we could not exclude the possibility that there may still be an association between progesterone levels and LBR, as the cutoff value for progesterone levels to affect LBR has been inconsistent in previous studies. Thus, we included progesterone levels in the multivariable models. The role of adding recombinant LH (r-LH) remains controversial, despite numerous clinical trials on this issue (39–43). The r-LH supplementation in this study depended on the physician preference, and the number of cycles with r-LH supplementation (n=71) was insufficient to draw conclusion. Future studies are needed to further reveal whether r-LH supplementation has an impact on clinical outcomes, especially with different groupings of LH levels. Besides, LH levels can vary and fluctuate significantly, and measured levels in the same individual may be significantly different one hour later, which may affect the applicability of the results. In addition, the relatively young age of the patients included in this study may have limited its applicability.

It is of most clinical importance to identify patients who could obtain better pregnancy outcomes after fresh embryo transfer. It seems that high LH levels predict benefits in implantation and live birth rates, even when only one or two embryos are available for transfer, which can be explained in several ways. First, successful implantation requires synchronization of endometrial receptivity and embryo development (44, 45). Previous studies have shown that pregnancy outcomes in FET cycles were significantly higher than those in fresh IVF cycles using GnRH antagonists (28, 46). These results suggest that endometrial receptivity is impaired during COH, an effect thought to be mediated by the negative impact of non-physiological levels of hormones on embryo-endometrium asynchrony (47–49). One possible explanation for our findings is that low LH may lead to embryo-endometrial asynchrony and defective placentation by binding to LHCGRs, which are widely expressed in the female reproductive tract (17, 18). It is speculated that high LH level on hCG trigger day without concomitant elevated progesterone may be associated with good endometrial receptivity. Second, while this works for most patients, a small proportion of patients may exhibit unexpected responses and may require individual evaluation. Individual differences in LH levels may be partly related to LH genotype, which cannot be assessed in our daily clinical practice. Third, many studies have shown an association between high E2 and adverse pregnancy outcomes in IVF cycles (35–38, 50–52). Therefore, we also included E2 levels on hCG trigger day in our regression models, which was not a predictor of LBR. Finally, patients with low LH actually had a better prognosis than those with higher LH, with younger age, better ovarian reserve and a higher rate of good-quality embryos transferred (**Supplementary Table 3**), and cycle outcomes including those related to oocyte and embryo quality did not show very significant differences between different LH stratifications (**Supplementary Table 2**), suggesting that embryo quality may not be responsible for the difference in implantation rates due to LH levels. However, it is unclear if there is a relationship between LH levels on the trigger day and clinical outcomes of FET cycles, which needs further research to find out. In PCOS patients, previous studies have suggested that excessive basal LH may be detrimental to clinical outcomes (11), which was similar to our finding that high basal LH negatively affected LBR in the multivariable model. Notably, on the conversely, high LH on hCG trigger day had a positive effect on LBR in the model.

In conclusion, there is a positive association between serum LH levels on hCG trigger day and LBR after fresh embryo transfer in both NOR and PCOS patients. LH may be a predictor of whether to schedule fresh embryo transfer in IVF cycles for better clinical outcomes. Further studies are required to uncover the underlying mechanism to better understand the relationship between LH and LBR in GnRH antagonist cycles.

## Data availability statement

The raw data supporting the conclusions of this article will be made available by the authors, without undue reservation.

## Author contributions

RZ and FL conceived and designed the study. All the authors analyzed and interpreted the data. LH contributed to the data collection. RZ performed the statistical analysis and wrote the article. FL and XQZ revised the article. All the authors approved the final version of the manuscript. All authors contributed to the article and approved the submitted version.
