# Supplementary material for: Association between serum LH levels on hCG trigger day and live birth rate after fresh embryo transfer with GnRH antagonist regimen in different populations
Source: Front Endocrinol (Lausanne). 2023 Jul 5;14:1191827. doi: 10.3389/fendo.2023.1191827 (PMC10354555; doi:10.3389/fendo.2023.1191827)
Supplement: Supplementary file 1 [file Table_1.docx]

**Supplementary Table 1** Univariable regression analysis for live birth after fresh embryo transfer for different populations.

| **Parameters** | **Normal responders**  **Crude OR (95% CI)** | **PCOS**  **Crude OR (95% CI)** | **Poor responders**  **Crude OR (95% CI)** |
| --- | --- | --- | --- |
| Age | 0.928 (0.909-0.947) | 0.971 (0.928-1.017) | 0.903 (0.863-0.945) |
| BMI  AFC  Basal FSH  Basal LH  Gonadotropin dose  Days of stimulation  **Antagonist dose**  Days of antagonist  r-LH supplementation (Yes vs. No)  Hormone levels on hCG trigger day  LH  Estradiol  Progesterone  Fertilization by ICSI vs. IVF | 0.997 (0.968-1.026)  1.037 (1.022-1.052)  0.988 (0.949-1.029)  1.032 (0.991-1.075)  0.999 (0.999-0.999)  1.030 (0.983-1.079)  **1.011 (0.887-1.152)**  0.996 (0.962-1.032)  0.855 (0.491-1.488)  1.042 (1.002-1.083)  1.000 (0.999-1.000)  0.757 (0.576-0.994)  0.987 (0.815-1.195) | 0.964 (0.921-1.010)  0.998 (0.970-1.028)  1.089 (0.979-1.211)  0.972 (0.940-1.005)  1.000 (0.999-1.000)  1.045 (0.976-1.119)  **1.123 (0.894-1.410)**  1.020 (0.958-1.087)  0.951 (0.272-3.326)  1.066 (1.011-1.124)  1.000 (0.999-1.000)  0.935 (0.573-1.525)  0.806 (0.553-1.175) | 0.987 (0.922-1.058)  0.957 (0.889-1.031)  1.012 (0.968-1.058)  0.998 (0.925-1.076)  1.000 (0.999-1.000)  1.046 (0.965-1.133)  **1.153 (0.844-1.573)**  1.051 (0.966-1.144)  0.411 (0.084-1.998)  1.008 (0.949-1.072)  1.000 (0.999-1.000)  0.939 (0.512-1.724)  0.626 (0.388-1.010) |
| No. of embryos transferred (2 vs. 1) | 2.754 (2.024-3.747) | 1.147 (0.653-2.013) | 2.180 (1.322-3.594) |
| Endometrial thickness*  Progesterone supplementation (Vaginal vs. IM) | 1.090 (1.046-1.135)  0.959 (0.802-1.146) | 1.161 (1.065-1.267)  0.995 (0.707-1.400) | 1.055 (0.962-1.157)  1.154 (0.795-1.677) |

*The day of hCG administration. OR, odds ratio; CI, confidence interval; BMI, body mass index; AFC, antral follicle count; IM, intramuscular.

**Supplementary Table 2** Cycle outcomes according to LH stratification on hCG trigger day in different populations.

| **Subgroups** | **Oocyte yield** | **Rate of normal fertilization** | **Rate of usable cleavage embryos** | **Rate of good quality embryos** |
| --- | --- | --- | --- | --- |
| Normal responders  < 25th  25-75th  > 75th  *P* value  PCOS patients  < 25th  25-75th  > 75th  *P* value  Poor responders  < 25th  25-75th  > 75th  *P* value | 6424/7098 (90.5)  11342/12532 (90.5)  4778/5317 (89.9)  0.372  1833/2416 (75.6)  3361/4490 (74.9)  1521/1994 (76.3)  0.401  800/892 (89.7) ^a^  1489/1594 (93.4) ^b^  512/552 (92.8) ^b^  0.003 | 4147/6091 (68.1)  7096/10598 (67.0)  2987/4469 (66.8)  0.261  1080/1706 (63.3)  2011/3224 (62.4)  859/1415 (60.7)  0.322  521/755 (69.0)  1011/1444 (70.0)  339/504 (67.3)  0.509 | 3484/4892 (71.2)  5888/8423 (69.9)  2534/3536 (71.7)  0.092  898/1305 (68.8)  1721/2513 (68.5)  754/1069 (70.5)  0.470  447/622 (71.9)  853/1188 (71.8)  294/401 (73.3)  0.833 | 3032/4085 (74.2)  5193/6986 (74.3)  2209/2941 (75.1)  0.659  811/1061 (76.4) ^a^  1587/1975 (80.4) ^b^  691/842 (82.1) ^b^  0.006  378/521 (72.6)  756/995 (76.0)  255/337 (75.7)  0.325 |

Variables are presented as number (percentage). ^a, b^ Different superscripts within the same line means statistically difference between subgroups.

The interquartile range (25th to 75th) for LH in normal responders was 1.62 to 3.86 mIU/ml; 2.25 to 5.68 mIU/ml in PCOS patients; and 2.14 to 4.72 mIU/ml in poor responders.

**Supplementary Table** **3** Patient characteristics according to LH stratification on hCG trigger day in normal responders.

| **Parameters LH levels on hCG trigger day stratified by percentiles** | | | | | |
| --- | --- | --- | --- | --- | --- |
|  | **< 25th (n=510)** | **25-75th (n=1028)** | **> 75th (n=511)** | ***P* value** | |
| Age  BMI  AFC  Basal FSH  Basal LH  Gonadotropin dose  Days of stimulation  Days of antagonist  r-LH supplementation  Hormone levels on hCG trigger day  LH  Estradiol  Progesterone  ICSI treatment  No. of embryos transferred  Single  Double  Rate of good-quality embryos transferred  Endometrial thickness*  Route of progesterone supplementation  Intramuscular  Vaginal + oral | 31.15 ± 4.39^a^  21.34 ± 2.94^a^  11.98 ± 5.98  6.96 ± 1.93^a^  4.72 ± 1.92^a^  2078 ± 846  9.94 ± 1.74  4.56 ± 2.63  21 (4.1) ^a^  1.10 ± 0.36^a^  2466.9 ± 1129.2  0.79 ± 0.34  144 (28.2)  47 (9.2)  463 (90.8)  809/973 (83.1) ^a^  10.62 ± 2.21  328 (64.3)  182 (35.7) | 32.27 ± 4.30^b^  21.82 ± 2.99^b^  11.35 ± 6.13  7.35 ± 2.10^b^  5.04 ± 2.20^b^  2063 ± 747  9.78 ± 1.81  4.66 ± 2.43  28 (2.7) ^a^  2.60 ± 0.63^b^  2454.4 ± 1181.4  0.76 ± 0.32  301 (29.3)  114 (11.1)  914 (88.9)  1562/1942 (80.4) ^a, b^  10.63 ± 2.12  631 (61.4)  397 (38.6) | 32.52 ± 4.17^b^  22.18 ± 2.91^c^  11.51 ± 6.09  7.53 ± 2.34^b^  5.45 ± 2.22^c^  1993 ± 777  9.81 ± 2.06  4.67 ± 2.44  3 (0.6) ^b^  6.09 ± 2.40^c^  2370.2 ± 1236.4  0.76 ± 0.30  143 (28.0)  64 (12.5)  447 (87.5)  753/958 (78.6) ^b^  10.74 ± 2.09  311 (60.9)  200 (39.1) | < 0.001  < 0.001  0.062  < 0.001  < 0.001  0.080  0.081  0.944  0.001  < 0.001  0.101  0.167  0.839  0.237  0.038  0.395  0.448 |  |

Continuous variables are presented as mean ± SD or median (Q1, Q3). Categorical variables are presented as number (percentage).

*The day of hCG administration. ^a, b, c^ Different superscripts within the same line means statistically difference between subgroups.
